# 969. The Importance of a Strong Handshake: Expanding Antimicrobial Stewardship to Engage Frontline Providers in Adult Medicine

**DOI:** 10.1093/ofid/ofac492.811

**Published:** 2022-12-15

**Authors:** Julia Sapozhnikov, Brian M Hoff, Jenna Adams, Maressa Santarossa, Sonali Kalathiya, Fritzie S Albarillo

**Affiliations:** Loyola University Medical Center, Chicago, Illinois; Loyola University Medical Center, Chicago, Illinois; Loyola University Medical Center, Chicago, Illinois; Loyola University Medical Center, Chicago, Illinois; Loyola University Medical Center, Chicago, Illinois; Loyola University Medical Center, Chicago, Illinois

## Abstract

**Background:**

The Joint Commission highlighted handshake stewardship (HS) as a leading practice in antimicrobial stewardship (AMS). However, intervention and outcomes data in adult populations are lacking. Additionally, limited human resources remain a barrier to widespread implementation of HS services. In February 2022, our antimicrobial stewardship program (ASP) expanded to include a HS service supporting adult internal medicine patients managed by hospitalist staff at an academic medical center. Here we aim to describe the interventions made to support and improve care in this population.

**Methods:**

This was a single-center, retrospective quality improvement initiative at a 547-bed academic medical center. Beginning in February 2022, the ASP structure expanded to include 2-week rotations between HS, general infectious diseases (ID) consult rounds, and whole house AMS (a combination of prospective audit and feedback and pre-authorization). During the HS rotation, all prescribed antimicrobials were prospectively reviewed Monday-Friday by the ID pharmacist and recommendations to improve quality, safety, and transitions of care were discussed in-person during daily rounds with hospitalists. Interventions were documented daily and data generated between February 1, 2022 and April 30, 2022 were reviewed to categorize the impact of the expanded service.

**Results:**

A total of 316 interventions were made for 101 unique patients over a 3-month period. We observed 286 accepted interventions (90.5%) during the intervention period. On average, the HS pharmacist reviewed 24 charts per day and spent 68 minutes in chart review, 16 minutes in rounds, and 23 minutes in other direct communication with providers per day. Table 1 and Figure 1 describe the distribution of interventions by type and indication. Table 2 quantifies the number of days broad spectrum antibiotics were avoided directly attributable to interventions. Since implementation, the HS service has contributed to an estimated cost savings of $73,470.19 for our hospital.

**Table 1.**
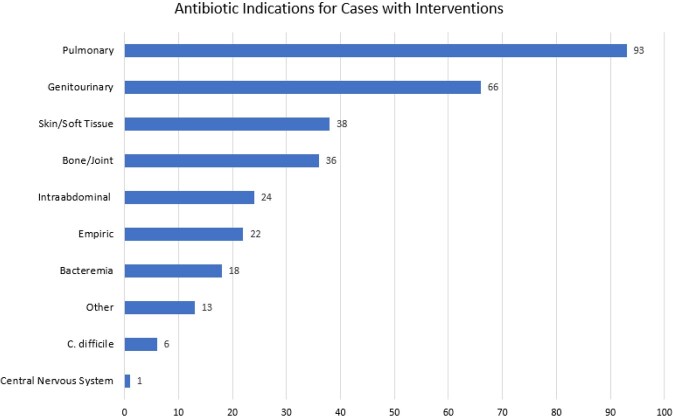
Intervention by Type

**Figure:**
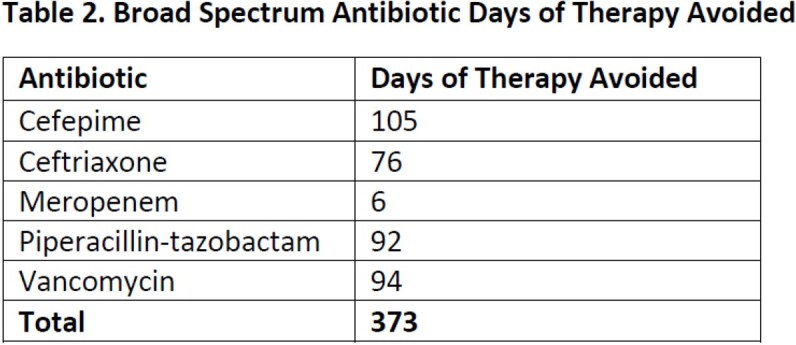

**Figure 1.**
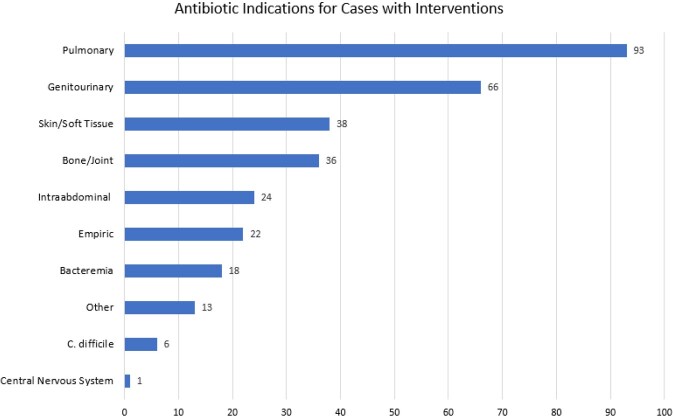
Antibiotic Indication for Cases with Interventions

**Conclusion:**

Implementation of HS in two adult internal medicine teams was associated with many interventions with high acceptance by hospitalist staff. Interventions led to tangible reductions in broad spectrum antibiotic days of therapy and cost.

**Disclosures:**

**Sonali Kalathiya, PharmD, MPH, BCIDP**, Blueprint Medicines: Spouse is employed by Blueprint Medicines|Blueprint Medicines: Stocks/Bonds.

